# Granulomas and Inflammation: Host-Directed Therapies for Tuberculosis

**DOI:** 10.3389/fimmu.2016.00434

**Published:** 2016-10-24

**Authors:** Hlumani Ndlovu, Mohlopheni J. Marakalala

**Affiliations:** ^1^Division of Immunology, Department of Pathology, University of Cape Town, Cape Town, South Africa; ^2^TB Immunopathogenesis Group, Division of Immunology, Department of Pathology, University of Cape Town, Cape Town, South Africa

**Keywords:** tuberculosis, *Mycobacterium tuberculosis*, granuloma, inflammation, host-directed therapy, drug-resistant TB, MDR-TB, tissue pathology

## Abstract

Tuberculosis (TB) remains a leading global health problem that is aggravated by emergence of drug-resistant strains, which account for increasing number of treatment-refractory cases. Thus, eradication of this disease will strongly require better therapeutic strategies. Identification of host factors promoting disease progression may accelerate discovery of adjunct host-directed therapies (HDTs) that will boost current treatment protocols. HDTs focus on potentiating key components of host anti-mycobacterial effector mechanisms, and limiting inflammation and pathological damage in the lung. Granulomas represent a pathological hallmark of TB. They are comprised of impressive arrangement of immune cells that serve to contain the invading pathogen. However, granulomas can also undergo changes, developing caseums and cavities that facilitate bacterial spread and disease progression. Here, we review current concepts on the role of granulomas in pathogenesis and protective immunity against TB, drawing from recent clinical studies in humans and animal models. We also discuss therapeutic potential of inflammatory pathways that drive granuloma progression, with a focus on new and existing drugs that will likely improve TB treatment outcomes.

## Introduction

One-third of the world population is latently infected with *Mycobacterium tuberculosis* (Mtb), the causative agent of tuberculosis (TB). About 5–10% of the latently infected population develops active TB and manifest clinical signs of the disease (1, 2). Active TB is estimated to cause 1.5 million deaths annually, making it one of the most deadly communicable diseases worldwide (3). Efforts to control and eradicate TB are hampered by coinfection with the human immunodeficiency virus (HIV) and emergence of drug-resistant bacteria. In 2014, 480,000 new cases of multidrug-resistant (MDR) TB were reported, and an estimated 9.7% of the MDR-TB patients have extensively drug-resistant (XDR) TB, characterized by resistance to second-line drugs and poor treatment outcomes (3). Therefore, there is an urgent need to develop new therapeutic drugs, an effective vaccine, and reliable diagnostic tests for latent disease in order to achieve the health targets of the newly adopted sustainable development goals (SDGs) aimed at ending the TB epidemic by 2030 (3).

The current standard first-line treatment regimen consisting of isoniazid, rifampicin, ethambutol, and pyrazinamide requires long duration of treatment (at least 6 months for first-line TB therapy and 18–20 months for MDR-TB therapy) to achieve sterilization of infection. Moreover, the toxic side effects of drugs may lead to non-compliance to treatment, thus, opening a window for development of drug resistance. Hence, novel treatment strategies are required to shorten the duration of treatment and improve treatment outcomes particularly in drug-resistant patients. The use of adjunctive host-directed therapies (HDTs) that seek to limit lung pathological damage and boost the host protective armory against Mtb infection is an attractive avenue requiring more research exploration (4–6). In this review, we discuss the role of the granuloma in the progression of TB disease and review HDTs that target pathways implicated in severe inflammation during TB disease.

## Initiation of Granuloma Formation: A Contribution of Innate Cells

**Figure 1:**
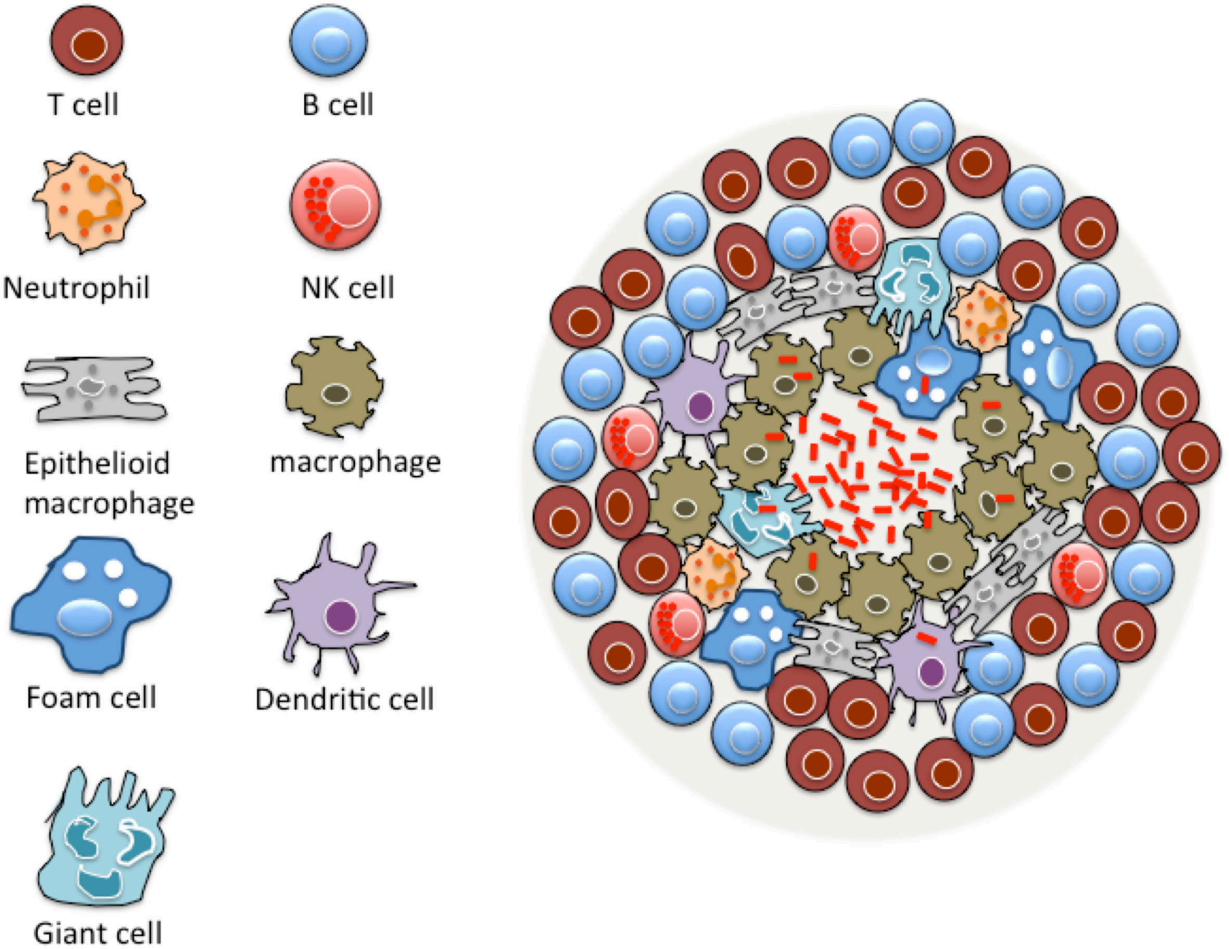
**Basic structure of TB granuloma**. A granuloma is a compact immunological structure rich with macrophages at the center. Macrophages can undergo specialized transformation differentiating into other cell types, such as epithelioid cells, multi-nucleated giant cells, and foamy macrophages that are accumulated with lipid droplets. A lymphocytic cuff that is largely comprised of B and T cells characterizes the periphery of the granuloma. Many other cell types are known to constitute a granuloma, including neutrophils, dendritic cells, natural killer (NK) cells, and fibroblasts.

A prominent pathological feature of TB is the formation of granulomas, driven by both bacterial and host factors in the lungs of infected patients (Figure 1) (6, 7). A granuloma is an organized and compact immunological structure rich with immune cells, such as macrophages, monocytes, dendritic cells (DCs), neutrophils, epithelioid cells, foamy macrophages, and multi-nucleated giant cells (8, 9). This initial structure is surrounded by a layer of lymphocytes giving it an organized solid structure (8, 9).

Early events that lead to granuloma formation were elucidated through a study by Davis and Ramakrishnan that utilized a transparent embryonic zebrafish model of *Mycobacterium marinum* (*M.m*) expressing fluorescent proteins (10). The study showed that bacteria freely replicate within macrophages and that mycobacteria direct recruitment and motility of uninfected macrophages into the surrounding areas of infected cells using the ESX-1/RD1 virulence locus (10). Moreover, mycobacterial virulence protein ESAT-6 induces the secretion of host matrix metalloproteinase-9 (MMP-9) by epithelial cells to drive the recruitment of new macrophages into the granuloma (11, 12). Studies conducted in humans and animal models have confirmed the crucial role of MMP-9 in pathogenesis of TB (7, 13–15). Infected macrophages departing the primary granuloma are involved in establishing secondary granulomas, thus, promoting early dissemination of infection (10). Mycobacterial cell wall glycolipid, trehalose 6,6′-dimycolate (TDM), is also known to trigger immunogenic responses that lead to granuloma formation in mice. The TDM-driven granulomatous response is initiated and maintained by pro-inflammatory cytokines (TNF-α and IL-6) and complement C5 and can be down modulated by lactoferrin (16, 17).

Macrophages within the granuloma develop into specialized cell types, such as epithelioid macrophages, foamy macrophages, and multi-nucleated giant cells that form after fusion of plasma membranes of multiple macrophages (18). Mycobacterial lipids, such as oxygenated mycolic acid, have been shown to trigger the differentiation of human monocyte-derived macrophages (MDM) into foamy macrophages *in vitro* (19). Murine macrophages infected with bacillus Calmette-Guérin (BCG) differentiate into foamy macrophages through a signaling process that is mediated by toll-like receptor 2 (TLR2) (20). Studies conducted in humans and murine models have associated foamy macrophages with necrotic regions of the granuloma (19). A recent study by Berg et al. showed that snapc1b zebrafish mutants, which were identified in a genetic forward screen by Tobin et al., were hypersusceptible to *M.m* infection due to early breakdown of granulomas that resulted in release of mycobacteria to a growth permissive extracellular milieu (21, 22). In addition, macrophages from snapc1b mutants displayed vacuolated morphology and had reduced speed of homeostatic migration due to accumulation of undigested debris in the lysosomes (22). This indicates that the migratory capacity and lysosomal functionality of macrophages is crucial for control of bacterial proliferation.

Dendritic cells are among the early arrivals at the site of infection where they partake in sampling of antigen by engulfing bacteria, bacterial products, and infected dying cells, leaving the site of infection and migrating *via* the lymphatic system to the draining lymph nodes where they possibly trigger acquired immunity (23). A study by Harding et al. showed that inflammatory DCs infected with BCG associated with bacteria-specific T cells, resulting in the formation of new multi-foci lesions, demonstrating their role in granuloma reformation (24). Moreover, Mtb can modulate DC function by impairing antigen presentation and migration to the draining lymph nodes, thereby delaying the development of the adaptive immune response required to arrest bacterial proliferation (23, 25).

Diverse myeloid cell subsets contribute to the granuloma and drive unique pathological outcomes during TB disease. Studies conducted in murine and non-human primate models showed that polymorphonuclear neutrophils (PMNs) are recruited early to the lung during virulent Mtb infection (26–29). PMNs either confer host protection by driving T-cell priming and granuloma formation or promote disease severity depending on the genetic background of experimental mice (30–34). PMNs have been implicated in lung damage during TB disease in studies conducted in mice (35) and humans (36). Kinetics studies in the lungs of C57BL/6 and 129S2 mice detected an accumulation of myeloid-derived suppressor cells (MDSCs) that drive inflammatory responses to Mtb and restrict selected T-cell responses (37, 38). MDSCs have also been identified in pleural effusions and blood of TB patients (39, 40).

The final step in granuloma formation is the arrival of T and B cells that form the lymphocytic cuff at the periphery of the granuloma giving it a solid intact structure (41–45). The arrival of Mtb-specific T cells in the lungs coincides with the arrest of bacterial proliferation 14–21 days after initiation of infection by producing TNF and IFN-γ that enhance macrophage microbicidal activity (1, 46–51). Mice deficient of CD4^+^ T cells are susceptible to Mtb infection and have decreased cellular infiltration to the lungs (52). Other T cell subsets involved in immune response to Mtb infections in mice and humans include γδ T cells, CD8 T cells and, CD1-restricted CD4/CD8 double negative T cells (53, 54). Development of an optimal Th1 and Th17 immune responses is critical for long-term control of Mtb infection (55, 56). However, an exuberant immune response may be detrimental to the host due to excessive tissue inflammation; hence, a more balanced Th1/Th2/Th17 immune response is more desired to drive host protection, while limiting tissue pathology.

## The Role of Inflammatory Pathways in Granuloma Dissociation and Disease Progression

**Figure 2:**
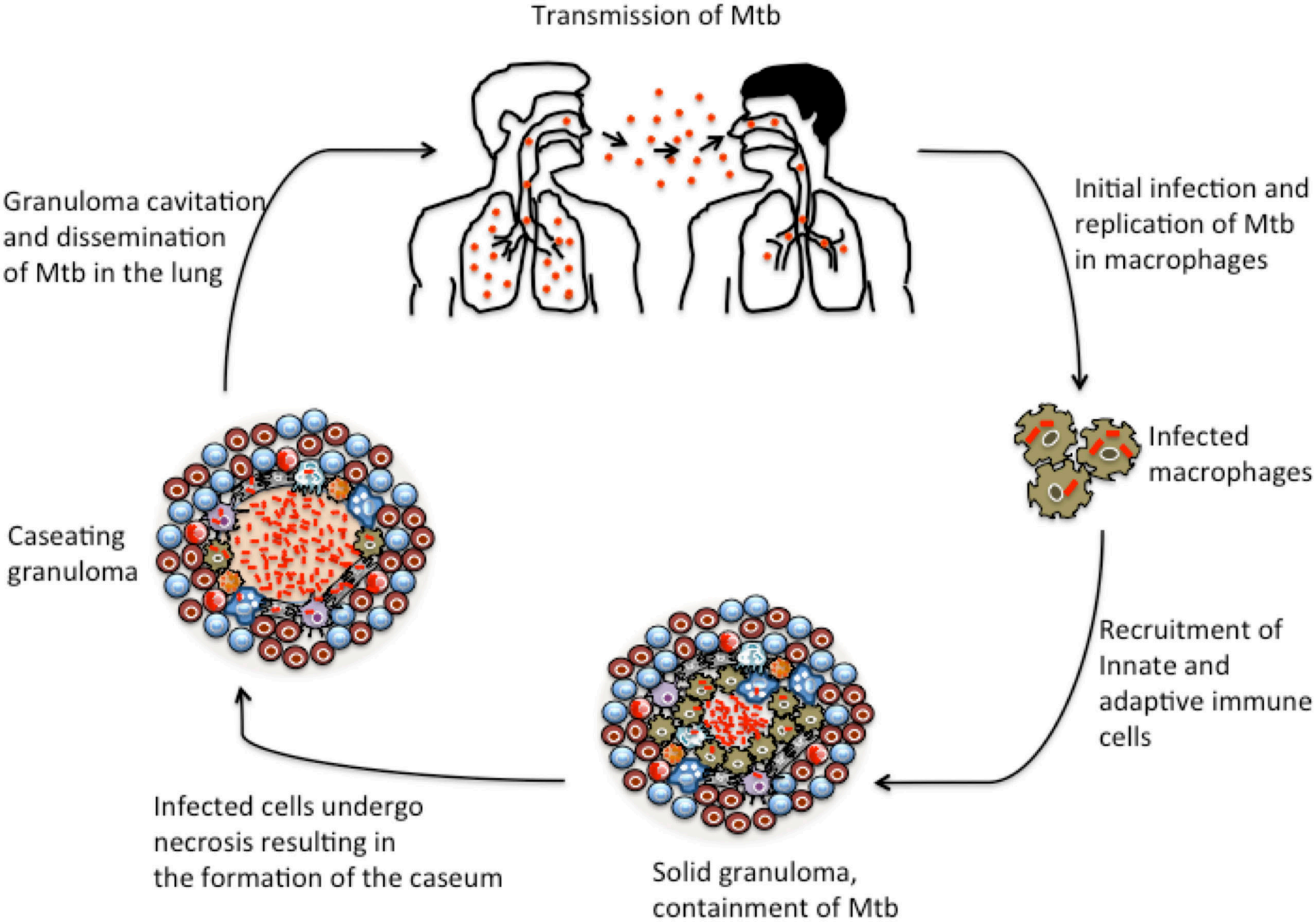
**Granuloma development during TB progression**. The initial stages of TB infection involve inhalation of *M. tuberculosis* bacilli into the lung and phagocytosis by resident alveolar macrophages. Mtb elicits local inflammatory response resulting in the recruitment of monocytes and macrophages and other innate immune cells to the site of infection. The macrophages further differentiate into other specialized cells, such as epithelioid macrophages, foamy macrophages, and multi-nucleated giant cells. Upon induction of adaptive immunity, the granuloma gets a peripheral lymphocytic cuff mainly characterized by B and T cells. This is the balanced solid state in which many granulomas persist and restrict the bacilli at their center. However, as the disease progresses, increased necrotic breakdown of granuloma cells leads to the accumulation of caseum, which may result in cavitation of granulomas. Ultimately, as the granuloma cavitates and collapses into the lung, Mtb bacilli are released into the airway.

As TB disease progresses, the granuloma may undergo complex remodeling events that result in the formation of a caseum, a cheese-like cellular structure containing necrotic material at the center of the granuloma that is rich in lipids and reportedly hypoxic. Analysis of human TB granulomas by genome-wide microarray found genes associated with lipid sequestration and metabolism to be highly expressed in caseous granulomas (57). Moreover, immunohistological studies revealed that cells surrounding the caseum expressed high levels of proteins involved in lipid metabolism, such as adipophilin, acyl-CoA synthetase long-chain family member 1, and saposin C (57). Destruction of the caseum results in liquefaction and eventually to granuloma cavitation, which permits the release of infectious Mtb into the airways where they are aerosolized in cough droplets and can facilitate bacterial transmission to new hosts (57, 58). The events that lead to granuloma collapse and spread of bacteria are depicted in Figure 2. Histological studies conducted in non-human primates (NHP) have elucidated the complexities of granuloma dissociation in relation to disease progression. A study by Lin et al. found that a discordance exists between the extent of granuloma destruction and the clinical state of disease in NHPs (59). Moreover, a subsequent study by Lin et al. showed that each individual granuloma within the same host behaves independently of others, suggesting that infected individuals have a heteregenous mixture of granulomas with varying immune profile and competence to control the bacteria (60). Hence, the fate of a few granulomas within the host seems to drive the majority of clinical morbidities associated with the disease (57, 60, 61).

Dissociation of the granuloma is driven by both host and mycobacterial factors that induce different cell-death modalities in infected cells. Infected granuloma macrophages can die either by apoptosis or necrosis. Apoptotic cell death seems to favor the host by keeping the bacteria encased within dead macrophages that can be readily phagocytosed by newly arriving uninfected macrophages. In contrast, necrotic cell death, which is characterized by the loss of cell membrane integrity, results in leakage of the bacteria into the growth permissive extracellular environment where the bacteria thrive by adopting a characteristic cording phenotype, making it difficult for the bacteria to be engulfed by new macrophages (62, 63). Host-derived TNF-α has been implicated in causing macrophage necrosis during Mtb infection (64, 65). Excessive production of TNF-α drives necrosis of infected macrophages through a programmed pathway called necroptosis that involves the activation of receptor activating protein 1 (RIP1) and RIP3 kinases *via* the production of reactive oxygen species (ROS) by the mitochondria (64, 65). TNF-α-induced macrophage necrosis is modulated by mitochondrial cyclophilin D *via* the formation of the mitochondrial permeability transition pore complex and the production of ceramide by the acid sphingomyelinase (65). Inhibition of cyclophilin D with alisporivir and inactivation of acid sphingomyelinase with an anti-depressant drug desipramine restored resistance to *M.m* infection in zebrafish (65).

Studies conducted in mice and zebrafish have shown that mycobacterial virulence factors can modulate macrophage necrosis by generating specific eicosanoids from arachidonic acid (AA) (21, 66). Virulent Mtb H37Rv strain induces the production of lipoxin A4 (LXA4) by 5- and 15-lipoxygenases to promote necrosis, while H37Ra mutants induced the synthesis of pro-apoptotic prostaglandin E2 (PGE2) by cyclooxygenase (COX) 1 and 2 and prostaglandin synthases (66–68). Mice lacking 5-lipoxygenase (ALOX5^−/−^) are more resistant to infection with Mtb H37Rv strain, indicating that LXA4 is detrimental to the host (21). The contribution of eicosanoid metabolites in susceptibility to mycobacterial infection was confirmed in a genetic screening study that aimed to identify mutant zebrafish that were hypersusceptible to *M.m* infection (21). The study identified leukotriene A4 hydrolase (LTA4H), an enzyme involved in the production of leukotriene B4 (LTB4) as a key determinant of host susceptibility to *M.m* infection (21). LTA4H mediates host susceptibility through two mechanisms that deregulate TNF-α activity: excess LTA4H causes overproduction of LTB4 that drives exuberant production of TNF, while low or absence of LTA4H induces the production of anti-inflammatory LXA4 that suppresses TNF activity leading to uncontrolled bacterial proliferation and macrophage necrosis (21, 64). Similar genetic variants encoding for low or high LTA4H expression have been identified in humans, and individuals who are homozygotes for low or high expression variants of LTA4H get severe TB meningitis with high mortality rate, while heterozygotes with intermediate LTA4H expression get protection (21, 64). Translation of these observations had huge implications for therapy as demonstrated in a Vietnamese cohort of TB meningitis in which adjunctive therapy with broadly anti-inflammatory glucocorticoids was beneficial to a group of patients with high LTA4H expression, while deleterious to patients with low LTA4H expression (21, 64).

A recent study by Marakalala et al. characterized protein and lipid signatures associated with granuloma progression by analyzing different granuloma types (intact, caseous, and cavitary granuloma) from tissue obtained from patients who underwent surgery due to severe lung disease (69). This study utilized laser-capture microdissection, mass spectrometry, and confocal microscopy to generate a molecular map of different regions of each granuloma type. The study identified over 3000 proteins that were differentially expressed between different regions of granuloma types (69). The study found more proteomic diversity within rather than between different granuloma types. Importantly, the proteomic profiles of caseous and cavitary centers were composed largely of pro-inflammatory pathways, including proteins that release pro-inflammatory eicosanoids from AA metabolism (69). In contrast, cellular regions found at the periphery of the granulomas displayed a more anti-inflammatory signature characterized by the abundance of pathways involved in normal cellular biogenesis and some enzymes involved in the synthesis of prostanoids (69). Together, these data suggest that spatial organization of inflammatory responses within the granuloma may determine the pathologic response to TB.

## The Granuloma: Is it Host Protective or Detrimental?

Initially, the granuloma was thought to be a solely host protective structure that “walls off” infecting bacteria (70, 71). However, we argue that the granuloma may represent a standoff between two warring camps: the immune response trying to kill the invading bacteria and the bacteria seeking to defend itself and establish a niche where it can thrive. Earlier histological studies conducted on tuberculous lungs obtained from autopsies in the pre-chemotherapy era chronicled a series of events associated with granuloma destruction (72, 73). Areas of the granuloma that had undergone caseation were correlated with increased bacterial load, while areas with intact granulomas had reduced bacterial numbers, thus, suggesting that the integrity of the granuloma is critical for control of bacterial proliferation (72, 73). This was further corroborated by findings in a zebrafish model, where it was shown that the onset of granuloma caseation is linked to uncontrolled bacterial proliferation (74). Moreover, mice deficient in TNF (48, 49, 75–79), IFN-γ (80), IL-12 (81), signal transducer and activator of transcription 4 (STAT4) (82), and myeloid differentiation primary-response protein 88 (MYD88) (83–85) had poorly formed granulomas and were highly susceptible to Mtb infection, thus, giving more credibility to the host-protective nature of granulomas. Although gene deficient mice are hypersusceptible to Mtb infection, the mechanism for their susceptibility may not depend on granulomatous responses but be driven by the impaired ability of infected macrophages to kill the bacteria (48).

Findings generated over the previous decade suggest that Mtb orchestrates the early events of granuloma formation for its own benefit, has adapted to life in the granuloma, and has developed an arsenal of strategies to evade host-offensive mechanisms. Recent technological advances have enabled us to chronicle the early events of granuloma formation in the context of innate immunity using dynamic imaging tools in a zebrafish model infected with *M.m* (10, 86). Mycobacteria coordinate granuloma formation by driving the recruitment and chemotactic motility of uninfected macrophages to the granuloma by deploying the ESX-1 secretory system encoded by the RD1 virulence locus (10). The recruitment of uninfected macrophages to the granuloma is further enhanced by the secretion of MMP-9 by epithelial cells activated by the mycobacterial virulence factor ESAT-6 (11, 12). MMP-9 has been identified as a biomarker for severe TB meningitis and is associated with increased mortality in human patients (13, 15). Interestingly, ESAT-6 has also been shown to induce apoptosis of infected cells *in vitro* (74, 87–89). The bacteria seem to be able to persist within the hostile environment of the granuloma as indicated by the propensity of newly infected macrophages to migrate to existing granulomas than avoid them in both zebrafish and mice with mature granulomas (90, 91). Thus, the ability of the bacteria to coordinate both recruitment and death of macrophages suggests that the bacteria are actively attempting to create an environment ideal for their dissemination and proliferation.

Mycobacteria have adapted to survive in the hostile environment by entering a state of non-replicating persistence characterized by slow growth and metabolic shift that reduces their susceptibility to environmental pressure (92). Moreover, the bacteria have developed strategies to survive the hypoxic environment encountered in caseous granulomas by inducing complex transcriptional networks like DosR regulon (93, 94) and angiogenesis to improve the transport of oxygen to the hypoxic granuloma (95). Suppression of granuloma associated angiogenesis by administration of VEGFA inhibitor reduced bacterial burdens and acted synergistically with first-line drugs to kill the bacteria (95). A study by Zhang et al. identified a set of genes (the “counteractome”) required by Mtb to survive the immunological stress mediated by CD4^+^ T cells in mice (96). CD4^+^ T cells starve Mtb of exogenous tryptophan, and deletion of *trpE* gene required for bacterial tryptophan biosynthesis rendered Mtb susceptible to the immunological stress (96). Interestingly, a combination of IFN-γ administration and inhibition of TrpE by a small molecule 6-FABA resulted in synergistic killing of Mtb *in vitro*, suggesting that inhibitors of the Mtb “counteractome” can be explored as drug candidates that could potentiate anti-mycobacterial immunity (96).

## Targeting Inflammatory Pathways to Develop Host-Directed Therapies for TB

One of the major challenges associated with current TB treatment protocols is the lengthy duration of therapy that causes poor compliance of patients, resulting in increasing cases of MDR- and XDR-TB and high mortalities among the treatment-refractory patients (6). In order to achieve the WHO goal to eradicate TB by 2050, we need new treatment strategies that will boost currently available drugs, shorten the duration of treatment, and ameliorate inflammation and tissue damage. One strategy that is showing greater promise of assisting in the fight against TB is the development of HDTs that could be used as adjunctive therapy in combination with the currently available standard treatment regimens. HDTs are aimed at augmenting the host’s immunological defense mechanisms and reducing excessive tissue pathology, culminating in improved clinical outcomes reflected in reduced duration of treatment and reduced morbidity and mortality (6). A wide range of HDTs are currently at varying stages of development and clinical testing, including repurposed drugs for diabetes, epilepsy, hypercholesterolemia, asthma, cancer, and arthritis (6).

The focus of this section is to discuss recent advances in the development of HDTs targeting inflammatory pathways associated with granuloma dissociation for which proof-of-principle has been achieved in animal models or clinical settings (Table 1). To have the full view of progress, made to date to develop and test HDTs, readers are referred to other extensive reviews (2, 5, 6, 97–99).

**Table 1:** **List of drugs inhibiting the inflammatory pathways implicated in granuloma dissociation with proven efficacy in animal models [adapted from Ref. (2, 6)]**.

Drug/compound	Potential target/pathway	Mechanism of action	Reported outcomes	Development stage
Etanercept	TNF-α	Blocks excess TNF-α	Reduced bacterial burdens and lung damage	Preclinical
Zileuton	ALOX5	Inhibits the synthesis of inflammatory eicosanoids by blocking the activity of lipoxygenases	Reduced bacterial burdens and increased survival of susceptible mice	Preclinical
Ibuprofen	Eicosanoid pathway	Limits inflammation by possibly modulating the production of anti-inflammatory eicosanoids	Increased survival of Mtb-infected mice and reduced tissue inflammation	Preclinical
Simvastatin	Cholesterol biosynthesis	Blocks the biosynthesis of cholesterol and augments autophagy and phagosome–lysosome maturation	Increased survival of Mtb-infected mice and ameliorated tissue pathology	Preclinical

### Targeting TNF to Limit Tissue Pathology

Although TNF is required to induce macrophage-killing activities (48, 49, 77), excessive- or low-TNF production is detrimental to the host causing augmented tissue pathology (21, 64, 65). Inhibition of TNF-α activity has been successfully used to treat inflammatory diseases, like rheumatoid arthritis (RA), Crohn’s disease, and psoriasis (5). Hence, blocking the activity of TNF has been explored as a potential host-directed therapy for TB. Treatment of Kramnik mice with a TNF-α inhibitor etanercept in conjunction with standard TB treatment reduced pulmonary bacterial burden and ameliorated lung pathology (100). However, the relapse of some of the mice treated with etanercept adjunctive therapy is of concern, albeit the relapse being lower compared with mice that received the standard treatment alone (100). The safety risks of using TNF-α inhibitors have also been demonstrated in human studies where patients receiving TNF-α inhibitors for chronic inflammatory disease like RA and Crohn’s disease can develop reactivation of TB disease (101, 102). Moreover, the presence of polymorphisms in LTA4H that skew TNF production is another confounding factor that needs to be addressed before considering the use of TNF-α inhibitors as adjunctive therapy in humans. Therefore, more studies are still required to improve the safety profile of TNF inhibitors.

### Pro-inflammatory Eicosanoids: Key Mediators of Granuloma Dissociation and Inflammation during TB

**Figure 3:**
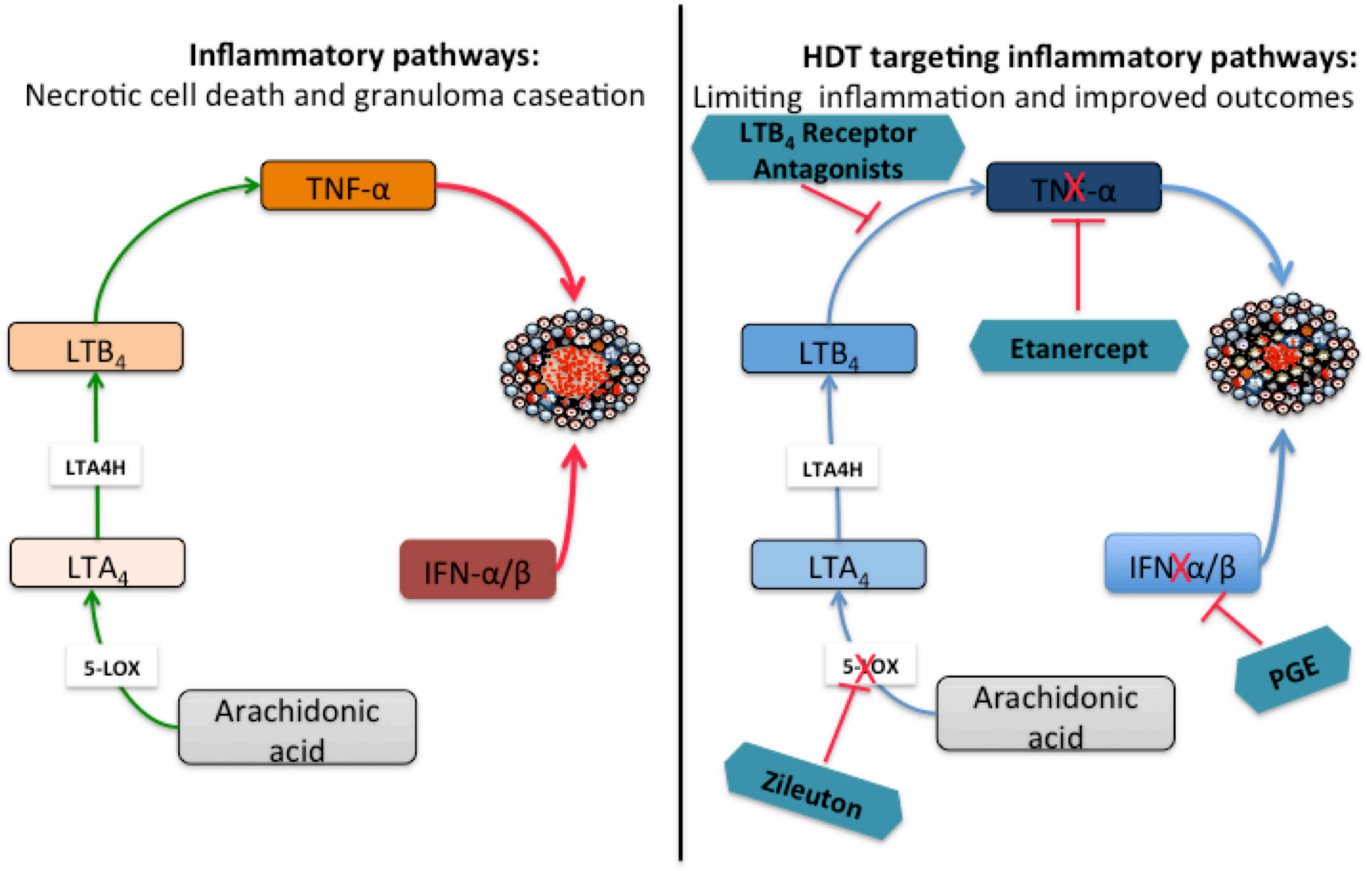
**Targeting inflammatory proteins as host-directed therapies (HDTs) for TB to ameliorate tissue inflammation**. Arachidonic acid (AA) is metabolized by 5-lipoxygenase (5-LO) to produce leukotriene A4 (LTA_4_), a substrate in the subsequent step that is catalyzed by leukotriene A4 hydrolase (LTA4H) to produce the highly inflammatory leukotriene B4 (LTB_4_). High levels of LTB_4_ drive excessive TNF-α production by macrophages that may trigger necrotic cell death. Necrotic cell death leads to granuloma dissociation resulting in the formation of caseums and eventually cavities after the necrotic center collapses into the airways. Type I IFNs block the production of host protective IL-1α and IL-1β *in vitro* and *in vivo* resulting in reduced production of PGE2. Targeting specific inflammatory proteins with chemical inhibitors or antibodies could be a possible HDT aimed at limiting tissue inflammation and improve treatment outcomes. The proof-of-principle for the interventions depicted in the schematic has been established in animal models.

As discussed above, pro-inflammatory eicosanoids have been implicated in disease progression by driving necrotic cell death and contribute to increased mortality of mice infected with Mtb. Recently, eicosanoids have been associated with the type I IFN response and contribute to the outcomes of both Mtb and influenza infections (103, 104). Studies conducted in mice and humans have demonstrated a deleterious role for type I IFNs to the host during Mtb infection (105–112). IFN-α/β have been shown to block the production of host protective cytokines IL-1α and IL-1β both *in vitro* using human and murine cells and *in vivo* using mice (113–115). Interestingly, IL-1α and IL-1β negatively regulate type I IFNs in murine and human macrophages and inhibit pro-bacterial effects downstream of IFN-β (103). IL-1-driven PGE2 production counter-regulates the function of type I IFN and is beneficial to the host as demonstrated in mice double deficient for IL-1R/IFNAR1 (103). This counter-regulatory mechanism has been shown to be at play in both human and murine cells infected with Mtb (103). The direct administration of PGE2 or blocking 5-lipoxygenase with zileuton rescued the highly susceptible IL-1-knockout mice from Mtb infection and reduced bacterial burdens (103). Therefore, manipulating the balance of eicosanoid by administering PGE2 or zileuton might offer an attractive adjunctive therapy for TB that could be used to improve the disease outcome (103). Interestingly, zileuton has already been approved for clinical use in asthma (103). The role of eicosanoids in driving granuloma dissociation and the potential benefits of targeting specific proteins as candidates for HDTs is summarized in Figure 3.

Inhibition of inflammatory eicosanoids with non-steroidal anti-inflammatory drugs (NSAIDs), such as ibuprofen, has been used as monotherapy for TB in mice with great success (116, 117). Treatment of C3HeB/FeJ (Kramnik) mice with ibuprofen increased survival of Mtb-infected mice by limiting severe tissue pathology and reducing bacterial load compared with untreated control mice (116). This study established a proof-of-principle for the use of NSAIDs as a host-directed adjunctive therapy for TB that can be used in combination with standard chemotherapeutic drugs.

### Targeting Lipids Modulates Inflammation and Improves Treatment Outcomes

The role of lipids in the dissociation of the granuloma has been demonstrated in a study that utilized genome-wide screening to determine the levels of genes involved in lipid metabolism in human TB lung tissues (57). This study found that genes involved in lipid metabolism were highly expressed in caseous granulomas, and this was further proven by immunohistological staining that confirmed the abundance of proteins involved in lipid metabolism in caseous regions of the granuloma (57). Mtb can utilize host cholesterol to maintain persistent chronic infection. Moreover, lipid bodies present within foamy macrophages have been shown to play a role in reactivation of latent TB disease (118, 119). Therefore, lipids have been targeted as possible host-directed therapy for TB disease using statins; competitive inhibitors of HMG-CoA reductase, an enzyme involved in the biosynthesis of cholesterol (120). Statins have been used clinically to treat patients with coronary disorders and hypercholesterolemia (121) and have been shown to possess immunomodulatory and anti-inflammatory properties (122, 123).

*In vitro* studies conducted using peripheral blood mononuclear cells (PBMCs) and MDM isolated from patients with familial hypercholesterolemia who underwent statin therapy were found to be resistant to Mtb infection compared with cells from healthy controls (124). In addition, treatment of mice with simvastatin prior to Mtb infection increased survival due to decreased lung bacterial load and improved pathological outcomes (124). The host-protective mechanism was shown to be mediated by the ability of statins to promote phagosomal maturation and autophagy (124). Statins have also been shown to enhance the bactericidal activity of first-line drugs *in vitro* using infected macrophages (125) and *in vivo* using a mouse model of chronic TB infection (126). A recent study by Dutta et al. showed that using simvastatin as an adjunctive therapy in combination with the first-line drug rifampicin shortened the duration of treatment in mice (127). Therefore, statins appear to be attractive candidates for host-directed therapy for TB. However, more work is still required to assess the safety, dosage, and efficacy of statins in humans infected with Mtb.

## Summary and Concluding Remarks

Tuberculosis still remains a huge public health problem with high mortality rate in infected individuals. Moreover, drug resistance has emerged as an enormous problem exerting more strain on health services. This has necessitated an urgent need to develop new drugs that not only target the pathogen but also helps to boost the host’s natural ability to fight the infection. The site that represents a standoff between the host and the pathogen as they seek to establish dominance is the granuloma; a key immunological structure composed of innate and adaptive cells of the immune system. Initially, the granuloma was thought to provide host protection by “walling off” the bacteria; however, evidence emerging from recent studies indicates that the granuloma may be manipulated by the mycobacteria to achieve its dominance in the host (70, 71, 128). Studies have demonstrated that virulence factors secreted by Mtb *via* the ESX-1 secretory system drive the recruitment of macrophages to the granuloma and influence the cell-death modality of infected macrophages by favoring necrosis mediated by the RIP1/RIP3 necroptosome (64, 65). Moreover, ESAT-6 has been shown to induce the secretion of MMP-9 by epithelial cells to further drive the recruitment of macrophages to the granuloma (11, 13–15).

The granuloma can undergo complex remodeling events driven by both bacterial and host factors resulting in some structural changes that coincide with TB disease progression. Studies have been conducted to understand the mechanism driving granuloma dissociation during TB disease (57, 69). Excessive TNF-α production has been implicated in the destruction of the granuloma in zebrafish (64, 65). Caseating regions of the granuloma have an abundance of proteins associated with lipid metabolism (57). Moreover, pro-inflammatory proteins, such as LTA4H, were highly expressed in caseous regions of the granuloma compared with cellular regions and associated with TNF-α expression (69). Hence, factors that drive granuloma dissociation have been explored as potential HDTs that could be used to improve treatment outcomes in conjunction with currently available standard treatment regimen. More studies are still required to further expand our knowledge of the molecular factors that sway the double-edged nature of the granuloma in favor of the host or the pathogen.

Blocking TNF-α with etanercept in the presence of first-line drugs proved beneficial to host by reducing bacterial load and limiting tissue pathology (100). Moreover, manipulating the balance of pro-inflammatory eicosanoids by directly administering PGE2 or using zileuton to block 5-lipoxygenase increased the survival of highly susceptible mice infected with Mtb (103). Finally, treatment of Mtb-infected mice with statins in the presence or absence of first-line TB drugs increased survival and reduced the duration of TB therapy (124, 127). These studies have established a proof-of-principle for the use of drugs that inhibit the pro-inflammatory pathways involved in granuloma dissociation in murine models. However, further studies are required to determine the safety, dosage, and efficacy of these drugs for use in humans. Challenges encountered with the administration of glucocorticoid steroids in patients with TB meningitis suggests that caution must be exercised in the roll-out of HDTs for TB and that perhaps personalized medicine might offer a better treatment approach.

Although not discussed in this review, a number of drugs currently used clinically to treat diabetes (115), cancer (95, 129), and hypertension (130) have shown some promise in anti-TB defense and can be repurposed for use as adjunctive therapy for the disease (2, 6). These will require more testing to ensure their efficacy and safety to use as adjunctive therapy for TB at the currently approved clinical dosages. To bolster our arsenal of adjunctive HDTs for TB, we need to amplify our efforts to identify potential new targets and improve our understanding of the complex processes that lead to disease progression at both molecular and systemic levels.

## Author Contributions

Both authors planned the manuscript content, analyzed the literature, and wrote the manuscript.

## Conflict of Interest Statement

The authors declare that the research was conducted in the absence of any commercial or financial relationships that could be construed as a potential conflict of interest.
